# Validation of an Enzyme-Driven Model Explaining Photosynthetic Rate Responses to Limited Nitrogen in Crop Plants

**DOI:** 10.3389/fpls.2020.533341

**Published:** 2020-09-25

**Authors:** Alamgir Khan, Zhiwei Wang, Kang Xu, Liyan Li, Lingchao He, Hanjian Hu, Genxuan Wang

**Affiliations:** Plant Physiology and Ecology Laboratory, Department of Ecology, College of Life Sciences, Zhejiang University, Hangzhou, China

**Keywords:** photosynthetic rate, leaf nitrogen content, crop plants, storage nitrogen, nitrogen absorption, CO_2_ concentrations, photosynthetic nitrogen use efficiency, nitrogen use efficiency

## Abstract

The limited availability of nitrogen (N) is a fundamental challenge for many crop plants. We have hypothesized that the relative crop photosynthetic rate (*P*) is exponentially constrained by certain plant-specific enzyme activities, such as ribulose-1,5-bisphosphate carboxylase/oxygenase (Rubisco), NADP-glyceraldehyde-3-phosphate dehydrogenase (NADP-G3PDH), 3-phosphoglyceric acid (PGA) kinase, and chloroplast fructose-1,6-bisphosphatase (cpFBPase), in *Triticum aestivum* and *Oryza sativa*. We conducted a literature search to compile information from previous studies on C_3_ and C_4_ crop plants, to examine the photosynthetic rate responses to limited leaf [N] levels. We found that in *Zea may*s, NADP-malic enzyme (NADP-ME), PEP carboxykinase (PCK), and Rubisco activities were positively correlated with *P*. A positive correlation was also observed between both phosphoenolpyruvate carboxylase (PEPC) and Rubisco activity with leaf [N] in *Sorghum bicolor*. Key enzyme activities responded differently to *P* in C_3_ and C_4_ plants, suggesting that other factors, such as leaf [N] and the stage of leaf growth, also limited specific enzyme activities. The relationships followed the best fitting exponential relationships between key enzymes and the *P* rate in both C_3_ and C_4_ plants. It was found that C_4_ species absorbed less leaf [N] but had higher [N] assimilation rates (*A*_rate_) and higher maximum photosynthesis rates (*P_max_*), i.e., they were able to utilize and invest more [N] to sustain higher carbon gains. All C_3_ species studied herein had higher [N] storage (N_store_) and higher absorption of [N], when compared with the C_4_ species. N_store_ was the main [N] source used for maintaining photosynthetic capacity and leaf expansion. Of the nine C_3_ species assessed, rice had the greatest *P_max_*, thereby absorbing more leaf [N]. Elevated CO_2_ (eCO_2_) was also found to reduce the leaf [N] and *P_max_* in rice but enhanced the leaf [N] and N use efficiency of photosynthesis in maize. We concluded that eCO_2_ affects [N] allocation, which directly or indirectly affects *P_max_*. These results highlight the need to further study these physiological and biochemical processes, to better predict how crops will respond to eCO_2_ concentrations and limited [N].

## Introduction

Insufficient levels of important chemical elements, such as nitrogen (N), can result in constraints on the metabolic fluxes required to produce enzymes in plants (Baudouin-Cornu et al., 2001). N resource allocation and its constraints specifically, have marked impacts on the assimilation rates of CO_2_ (*A_rate_*) (Von Caemmerer, 2000). The Michaelis-Menten equation (MME) (Michaelis and Menten, 1913) was derived to describe the relationship between metabolism and limiting resources (Carr et al., 1997; Aguiar-González et al., 2012), and it can also explain a plants’ ability to live and grow for long periods when its resources are limited. Generally, C_3_ and C_4_ photosynthesis represents a balancing act between the Calvin-Benson cycle enzymes and N resource allocation. This shows that crop plants optimally allocate their nutrients to obtain a “functional equilibrium” for fitness. Therefore, resource availability or the demands of metabolic scaling, depend on the capacity of the species (Glazier, 2018) and of the plant leaves to efficiently utilize their resources (P’yankov et al., 2001). The availability of limited resources and the demand of each species are scaled differently, creating variations in the leaf traits that govern leaf economy. The leaf economic traits that are related to the carbon (C) and N concentrations of the plant have a strong influence on the leaf photosynthetic traits, both among and within species (Hu et al., 2015). However, as greater growth rates require enhanced N levels, N can become the more limiting nutrient in soils of terrestrial ecosystems (Sardans and Peñuelas, 2015). Leaf traits such as N allocation and photosynthetic capacity, may differ significantly among various crop plants; hence, an improved understanding of the various scalings of leaf trait relationships would be valuable for the fields of ecology, plant biology, and crop science. For example, the various scalings of the leaf traits related to leaf functional traits, including the photosynthetic rate, N concentration, and CO_2_ concentration, are the main drivers of leaf trait variation. The study of leaf trait variations in different groups of plants has previously been the focus when trying to understand plant adaptations to limited N concentrations and low and elevated CO_2_ concentrations. Based on these ideas, we developed a novel enzyme-driven model (EDM) that hypothesizes that the photosynthetic rate has an exponential relationship with basic enzymes, and that the photosynthetic rate is dependent on effective N sources.

This investigation focused on the effects that the N content of leaves has on the photosynthetic rates of C_3_ and C_4_ plants. N is an essential plant nutrient in both agricultural and natural environments as every plant species requires it for growth (Evans, 1983; LeBauer and Treseder, 2008), and if it is limited, there may be negative consequences such as reduced crop yields (Xu et al., 2012). N significantly affects growth, because a large N investment is required for the assimilation of C (Evans, 1989; Hohmann-Marriott and Blankenship, 2011), and consequently, leaf N determines a plant’s growth potential. N is one of the main elements in the photosynthetic apparatus and understanding the relationship between photosynthesis and leaf N is critical for optimizing C production and identifying the mechanisms that regulate photosynthesis. Plants invest a huge amount of N into their photosynthetic machinery (Ghannoum et al., 2005), and so, leaf N has a positive correlation with photosynthesis (Paponov and Engels, 2003) and various N components in the allocation of leaf N (Uribelarrea et al., 2009). Evans (1989) revealed that the relationship between leaf N and photosynthetic capacity varied among the different types of plants. When integrating information on the anatomy with the mechanical properties, the nutrient and light availabilities were found to possibly scale leaf traits, meaning that they could alter the properties of the leaf morphology and structure (Onoda et al., 2008). The photosynthesis rates and N concentrations increase when moving from the shade to the sun (Niinemets et al., 2015), as light is important for the partitioning of N in photosynthesis (Ishimaru et al., 2001; Yamori et al., 2011); hence, light absorption influences the photosynthetic transport chain and further enhances yields and photosynthetic productivity (Ye et al., 2013). Therefore, both nutrient and light availabilities affect the activity of Rubisco and PEP carboxylase (PEPC) (Usuda, 1984; Meinzer and Saliendra, 1997). Various other leaf traits are also affected in N-limited conditions (Makino and Ueno, 2018). Consequently, N limitations affect the photosynthetic machinery (Evans, 1983), reduce chloroplast size (Laza et al., 1993; Bondada and Syvertsen, 2005; Kelly, 2018), and markedly influence plant growth and nutrient cycles (Osnas et al., 2018). Strong correlations were found between the limiting enzymes and leaf N content in relation to photosynthesis under low and high partial pressures of CO_2_ (Makino et al., 2003).

The rate of CO_2_ assimilation in relation to the N content is known as the photosynthetic nitrogen use efficiency (PNUE). Different molecular and physiological factors cause variations in the PNUE (Rotundo and Cipriotti, 2017), and as a result, there are large differences between plant species. Accordingly, C_4_ plants have a 50% greater photosynthetic rate than C_3_ plants with the same N concentration (Evans and von Caemmerer, 2000). Consequently, a higher NUE was found in the C_4_ pathway than in the C_3_ pathway (Kelly, 2018). The increased NUE of the C_4_ species compared with that in the C_3_ species shows that the availability of N had a positive role in their evolution (Vogan and Sage, 2011). Furthermore, C_4_ plants exhibit two times higher Rubisco activity, compared with C_3_ plants (Sage, 2002); hence, lower Rubisco concentrations may enhance the photosynthetic rates of C_4_ plants (Makino et al., 2003). Due to the reduction in photorespiration, C_4_ plants show higher photosynthesis rates (Sage and Pearcy, 1987). In addition, higher N uptake capacity has been correlated with photorespiration (Dellero et al., 2015; Busch et al., 2018), raising the question as to whether CO_2_ concentrations affect plant N uptake.

To investigate the responses of C_3_ and C_4_ crop plants to atmospheric CO_2_ (atmCO_2_), an in-depth study at the leaf level is required. However, a previous investigation on elevated CO_2_ (eCO_2_) showed positive physiological feedback responses in crops (Sakai et al., 2006). Furthermore, CO_2_ plays an integral role in plant photosynthesis, thereby affecting plant metabolism. An improved understanding of the plant responses when there is limited N to atmCO_2_ is required in order to predict future changes in their leaf photosynthetic properties (Osada et al., 2010), as well as their physiological and morphological changes (Ainsworth and Long, 2005), and photosynthetic capacities (Ghannoum et al., 2000). To understand the total N content and the N allocations to the photosynthetic machinery, which contribute to the diversity of various photosynthetic capacities, a great deal of research is required. The reduction in leaf N mostly aggravates photosynthetic acclimation to eCO_2_ (Halpern et al., 2019), while low N availability reduces the photosynthetic capacity by reducing the C assimilation proteins, as well as Rubisco (Cohen et al., 2019b). Finally, both low leaf N and eCO_2_ may have adverse impacts on the expression of Rubisco, which stimulates eCO_2_ (Cohen et al., 2019a). Global atmCO_2_ concentrations have been increasing, and the magnitude of these enhancements due to CO_2_ enrichment, varies with species and other limiting environmental conditions. Limited N availability may constrain the stimulation of plant growth by eCO_2_ (Bloom et al., 2014), which raises the question of how atmCO_2_ affects and constrains leaf N content and the response of photosynthesis in crops. To understand the responses of the crop photosynthetic rates to low and elevated [CO_2_], crop plants receiving N at the leaf level and the responses of their half-photosynthesis constants (Kp) to the effective limited N sources were questioned.

The Calvin-Benson cycle carries out C assimilation, which produces carbohydrates from atmCO_2_ using ATP and NADPH in photochemical reactions (Calvin, 1989; Benson, 2002). The Calvin cycle of C_3_ plants fixes the C in mesophyll cells, and the Rubisco enzyme further catalyzes it. In C_4_ plants there are two types of cells, known as mesophyll cells and bundle sheath (BS) cells, and they fix CO_2_ with phosphoenolpyruvate, catalyzed by PEPC, which has a higher affinity for CO_2_ than Rubisco. The photosynthetic rate changes during leaf development, which explains why such changes occur *via* the activities of the Calvin cycle enzymes. In general, the activities of the Rubisco enzyme decrease at a faster rate during leaf senescence (Nakano et al., 1995; Crafts-Brandner et al., 1998; Ishizuka et al., 2004). Like C_3_ plants, the main CO_2_ limitations occur due to Rubisco in C_4_ plants (Von Caemmerer et al., 1997). Increasing the amount of N enhances the PEPC activities relative to Rubisco (Sugiyama et al., 1984). The higher expression of Rubisco in N-limited plants shows that a reduction in Rubisco could reduce leaf N, due to the reallocation of the N to younger leaves in the N-limited plants (Nie et al., 1995). In addition, allocations of leaf N to the PEPC and PEPC to Rubisco were reduced under limited N conditions (Sage et al., 1987). Our EDM identified a relationship between photosynthesis and important plant enzymes, such as ribulose-1,5-bisphosphate carboxylase/oxygenase (Rubisco), NADP-glyceraldehyde-3-phosphate dehydrogenase (NADP-G3PDH), 3-phosphoglyceric acid (PGA) kinase, and chloroplast fructose-1,6-bisphosphatase (cpFBPase) in wheat and rice (C_3_ plants). While in C_4_ plants, NADP-malic enzyme (NADP-ME), PEP carboxykinase (PCK), Rubisco (*Zea mays* for C_4_ plant), and Rubisco and PEPC (*Sorghum bicolor*, C_4_ plant) were shown to be involved with photosynthesis. In this published literature study, we selected major C_3_ and C_4_ crop plants, and predicted that their photosynthetic rates would exponentially increase with the plant enzyme activities. In addition, we predict that the log-photosynthesis rates were dependent on the resource levels at the leaf level. We hypothesized that along with the key enzymes and resources, other factors (leaf trait variation, PNUE, low and high CO_2_, and N allocation) also played important roles in plant photosynthesis.

## Materials and Methods

### Data Sources

A search of the published literature from 1980 to 2018 was conducted to identify studies on the photosynthetic responses of important C_3_ and C_4_ crop plants to limited plant-specific enzyme activities, limited leaf N content, and partial pressures of [CO_2_]. We searched for the following six key terms alone using the ISI Web of Science and Google Scholar: “leaf N,” “specific enzymes,” “low partial pressure of [CO_2_],” “high partial pressure of [CO_2_],” “low and high partial pressure of [CO_2_],”, and “assimilation rate.” We then searched for the six key terms again using the ISI Web of Science and Google Scholar, but this time, each in combination with the following three terms individually: “C_3_ photosynthesis rates,” “C_4_ photosynthesis rates,” and “C_3_ and C_4_ photosynthesis rates.” This yielded 12 and 48 studies, respectively. The published articles identified with these search terms were then further screened using the following principles: 1) the study organisms were C_3_ or C_4_ crop species of interest; 2) the responses of photosynthesis in the C_3_ and C_4_ plants were measured; 3) the response variables under limited leaf N were reported; and 4) the responses of photosynthesis to the current and elevated partial pressures of the (CO_2_) were reported in figures and tables. As a result of this screening procedure, we ultimately selected 47 published articles for the analysis. Fifteen of the articles involved C_4_ crops: five on *Sorghum bicolor* (*sorghum*), and ten on *Zea mays* (maize). Fourteen of the articles involved C_3_ cereal crops: six for *Triticum aestivum* (wheat), seven for *Oryza sativa* (rice), and one for *Hordeum vulgare* (barley). Twelve of the articles involved C_3_ dicotyledonous crops: four for *Glycine max* (soybean), six for *Helianthus annuus* (sunflower), and two on *Solanum tuberosum* (potato). Finally, there were four publications relating to C_3_ trees: one for *Citrus sinensis* (Citrus orange), one for *Malus domestica* (apple), and two for *Prunus persica* (peach). Furthermore, 30 of the publications were related to photosynthesis, 40 to leaf N (leaf N content, 20 publications; N limitation, four publications; N response, four publications; N distribution, two publications; and N availability, 10 publications), three to enzyme activities, one to partial pressure of [CO_2_], and six to NUE.

**Figure 1:**
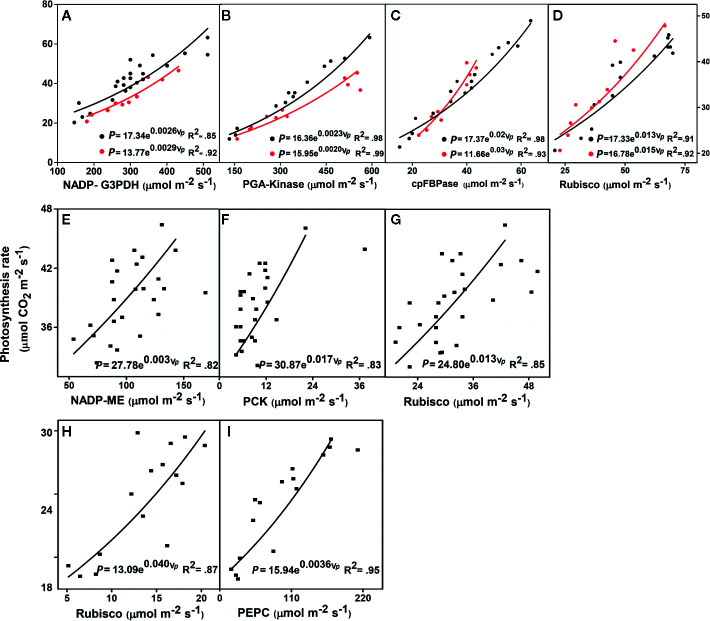
Relationships between the leaf photosynthesis rates and key enzyme activities. **(A–D)** *Triticum aestivum* (wheat, black circles) and *Oryza sativa* (rice, red circles), both C_3_ plants. **(E–G**) *Zea mays* (maize), a C_4_ plant. **(H, I)** *Sorghum bicolor* (sorghum), a C_4_ plant. NADP-G3PDH, NADP-glyceraldehyde-3-phosphate dehydrogenase; PGA-kinase, 3-phosphoglyceric acid kinase; cpFBPase, chloroplast fructose-1,6-bisphosphatase; Rubisco, ribulose-1,5-bisphosphate carboxylase/oxygenase; NADP-ME, NADP-malic enzyme; PCK, PEP carboxykinase; PEPC, phosphoenolpyruvate carboxylase; P, photosynthetic rate; V*_p_*, enzyme activities. *P*-values arising from one-way ANOVA (Tukey test) are presented, significance at (*P* < 0.05). All parameter values are given in **Table 1**. The data for *Triticum aestivum* and *Oryza sativa* were taken from Sudo et al., 2003, for *Zea mays* were from Yabiku and Ueno, 2017, and for *Sorghum bicolor* were from Makino and Ueno, 2018. The complete detailed mentioned in *Materials and Methods*, *Data Sources*.

**Figure 2:**
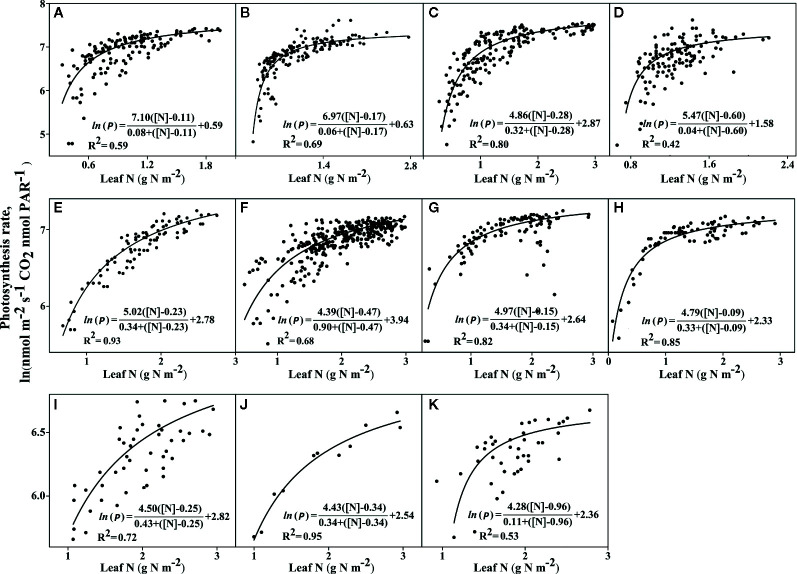
The photosynthetic rates in light-saturated conditions, which are dependent on leaf nitrogen [N] levels. Two C_4_ plants: **(A)** *Sorghum bicolor* and **(B)** *Zea mays*, and nine C_3_ plants **(C)** *Triticum aestivum*, **(D)** *Oryza sativa*, **(E)** *Hordeum vulgare*, **(F)** *Glycine max*, **(G)** *Helianthus annuus*, **(H)** *Solanum tuberosum*, **(I)** *Citrus sinensis*, **(J)** *Malus domestica*, and **(K)** *Prunus persica*. All parameters were determined using a best-fit non-linear regression line, and the R^2^ and *P*-values were determined using one-way ANOVA (Tukey’s test). Statistical significance was defined as *P* < 0.05. The sources for the data and references presented are given in **Dataset_S1**. The complete detailed mentioned in *Materials and Methods*, *Data Sources*.

**Figure 3:**
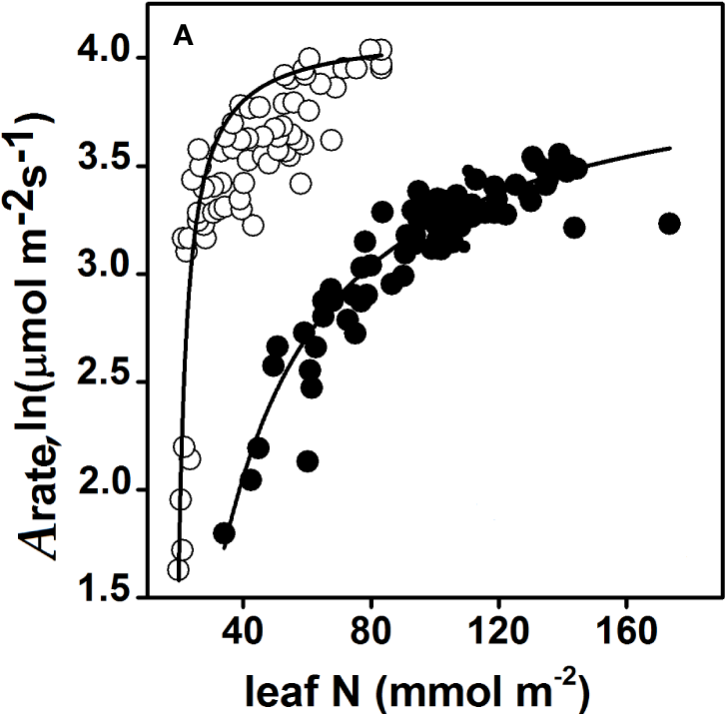
Response of the CO_2_ assimilation rate (*A*_rate_) to the same nitrogen content per unit leaf area in *Zea mays* (maize) (C_4_, white circle) and *Oryza sativa* (rice) (C_3_, black circle) plants. For the response of the nitrogen assimilation rate, *A*_rate_ = 4.12, leaf N = 18.55, and R^2^ = 0.78 (maize); *A*_rate_ = 4.05, leaf N = 18.75, and R^2^ = 0.91 (rice). The data collected for maize were from Sinclair and Horie, 1989 and Wong et al., 1985, and the data collected for rice were from Cook and Evans, 1983 and Makino et al., 1988. All responses were significant, and *P*-values arising from one-way ANOVA (Tukey’s test) analyses are presented. Statistical significance was defined as *P* < 0.05. The complete detailed mentioned in *Materials and Methods*, *Data Sources*.

**Figure 4:**
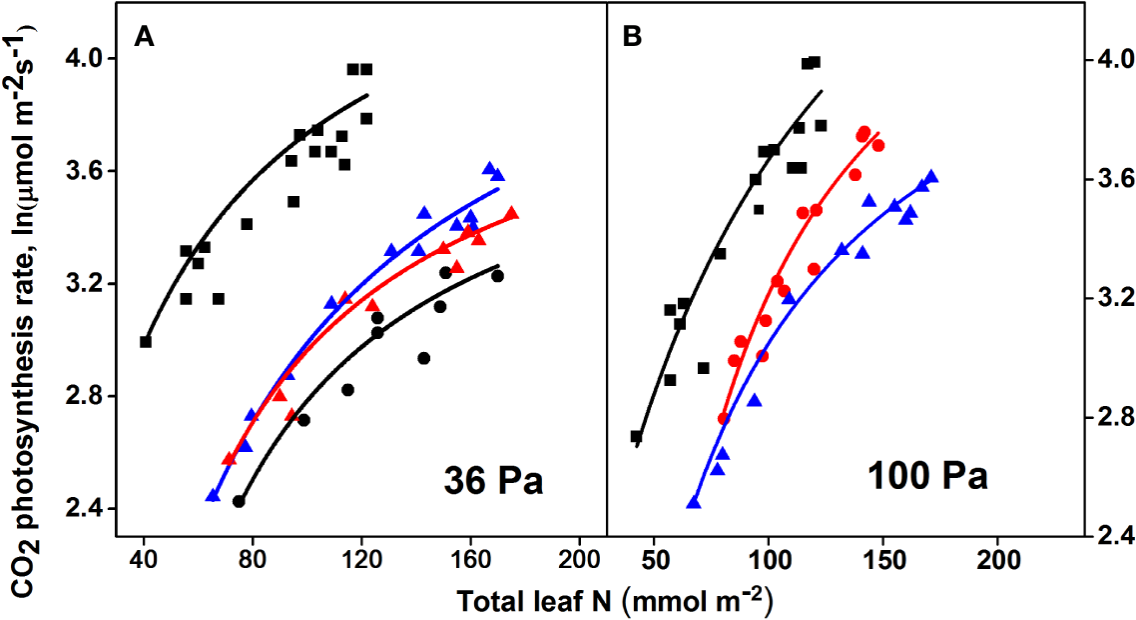
The response of the logarithmic-maximum photosynthetic rates to current (36 Pa) and elevated partial pressure (100 PaCO_2_) when the total leaf N was limited in the young leaves of C_3_ and C_4_ plants: **(A)** *Zea mays* (black squares, C_4_), *Oryza sativa* (blue triangle, C_3_), *Spinacia oleracea* (red triangle, C_3_), and *Phaseolus vulgaris* (black circles, C_3_); **(B)** *Zea mays* (black squares, C_4_), anti-rbcS 77 which is *rbcS* antisense rice with 65% wild-type Rubisco (red circles, C_3_), and *O. sativa* (blue triangles, C_3_). One-way ANOVA represents statistical differences (*P* < 0.05) by Tukey’s test and best R^2^. All the parameters are given in **Table 2**. The data were collected from Makino et al., 2003, and at 36 Pa for rice were from Makino et al., 1997 and at 100 Pa for rice including *rbc*S antisense rice from Makino et al., 1997. The complete detailed mentioned in *Materials and Methods*, *Data Sources*.

All the data for our analysis were obtained from the figures and tables of the 47 papers by using the software GetData Graph Digitizer 2.22. For each dataset, we used one-way ANOVA and Tukey tests to assess each of the parameters (mentioned in figures, tables, and statistical analysis), using Origin 9.0 software (Data analysis and Graphing software). For all figures, the sources for the data (see **Dataset_S1**) and references presented are given in Figure legends and **Dataset S1**. We analyzed the relationships between the enzyme activities (µmol m^-2^ s^-1^) and the photosynthetic rates (µmol CO_2_ m^-2^ s^-1^) in the young leaves of various C_3_ (wheat and rice) and C_4_ (maize and sorghum) plants. The methods for quantifying the roles of the enzymes *via* the photosynthetic rates are explained in the subsection section “*Model Background*”; the literature and data utilized for this concept can also be extracted from **Figure 1** and **Table 1**. We also tested the goodness of fit statistics (R^2^ and Akaike information criterion, AIC) of exponential and linear model for photosynthetic rate and various enzyme activities (see **Supplementary Material** for **Supplementary Table 1**). Additionally, we select data as a line-symbol for photosynthesis and leaf N content (0.05, 0.2, 0.4, or 0.6 g N) relationships in *Sorghum bicolor* (see **Supplementary Material** for **Supplementary Figure 1**); sources were obtained from Makino and Ueno (2018). The data analyzed to determine the relationship between the light-saturated logarithmic-photosynthetic rates (nmol m^-2^ s-1 CO_2_ nmol PAR^-1^) and the N content (g N m^-2^) in the leaves of the C_3_ and C_4_ plants can be found in **Figure 2** and **Dataset S1**. Here, we used only the logarithmic-photosynthetic rates to study the effects of the leaf N content on the leaf photosynthetic capacity. The comparisons between the logarithmic-assimilation rates (µmol m^-2^ s^-1^) and the same amount of leaf N content per unit area (mmol m^-2^) in the C_3_ (rice) and C_4_ (maize) plants were also determined (**Figure 3**); the sources were obtained from Evans and von Caemmerer (2000). Ignoring *P_0_* in Eq. 3 (a detailed explanation is given in “*Model Background*”) allowed for the responses of the logarithmic-assimilation rates at the leaf level to the same amount of leaf N content per unit area to be identified. A comparison between the C_3_ and C_4_ plants at 36 and 100 Pa, their logarithmic-photosynthesis rates (µmol CO_2_ m^-2^ s^-1^), and their leaf N content (mmol m^-2^) in the young leaves were obtained and digitized from the figures (**Figure 4**, **Table 2**), and the specific data sources are given in **Dataset S1**. Additionally, we further tested the line-graph analysis for positive correlation between photosynthesis rates and leaf N content by showing that N deficiency causes areduction in the photosynthetic rate and intercellular CO_2_ concentrations (Ci) (see **Supplementary Figure 2** in **Supplementary Material**); the sources were obtained from Zhao et al. (2005). Furthermore, Eq. 3 also allowed for the identification of the plant responses at the leaf level to the eCO_2_ concentrations, which are essential for predicting the structural changes and biochemical dynamics in plants.

**Table 1:** Effect and the relationships between the photosynthetic rates (µmol CO_2_ m^-2^ s^-1^) and enzyme activities (µmol m^-2^ s^-1^) in the young leaves of C_3_ and C_4_ plants.

Parameters	Photosynthesis rate (*P*)	Enzyme activity (*v_p_*)	R^2^
***Triticum aestivum* (wheat)**			
NADP-G3PDH	17.34 (2.04)	0.0026 (0.0003)	0.85
PGA-kinase	16.36 (1.59)	0.0023 (0.0001)	0.98
cpFBPase	17.37 (0.906)	0.0203 (0.0010)	0.98
Rubisco	17.33 (2.17)	0.0137 (0.0019)	0.91
***Oryza sativa* (rice)**			
NADP-G3PDH	13.77 (2.02)	0.0029 (0.0004)	0.92
PGA-Kinase	15.95 (0.65)	0.0020 (0.00009)	0.99
cpFBPase	11.66 (2.45)	0.0336 (0.00536)	0.92
Rubisco	16.78 (1.21)	0.0159 (0.00157)	0.92
***Zea mays* (maize)**			
NADP-ME	27.78 (1.73)	0.0033 (0.00057)	0.82
PCK	30.87 (0.79)	0.017 (0.00215)	0.83
Rubisco	24.80(1.66)	0.013 (0.002)	0.85
***Sorghum bicolor* (sorghum)**			
Rubisco	13.09 (1.04)	0.0406 (0.00508)	0.87
PEPC	15.94 (.62)	0.0036 (0.000297)	0.95

P-values arising from one-way ANOVA (Tukey test) are presented, significance at (P < 0.05).

Standard errors are given in parentheses.

**Table 2:** Variations in the logarithmic-photosynthesis rates (ln*P*_max_) of C_3_ and C_4_ species, at 36 and 100 partial pressure, under limited leaf N content (R – R_0_).

	Current 36 partial pressure(Pa) [CO_2_]				Elevated 100 partial pressure (Pa) [CO_2_]		
Parameters	*Zea mays* ^b^	*Oryza sativa* ^a^	*Spinacia oleracea* ^a^	*Phaseolus vulgaris* ^a^	*Zea mays* ^b^	Anti-*rbc*S 77^a^	*Oryza sativa* ^a^
P_max_	4.72	4.58	4.15	3.98	5.31	4.73	4.45
R-R_0_	9.7	12.68	19.37	26.31	26.09	25.54	7.98
Kp	29.19	46.83	32.40	31.87	51.63	28.45	32.35
R^2^	0.96	0.97	0.99	0.96	0.96	0.97	0.99

Significant difference; P < 0.05, Kp, half Michael’s constant; ^a^C_3_ plant; ^b^C_4_ plant; (R – R_0_), limited leaf nitrogen concentration; (lnP_max_), maximum logarithmic-photosynthesis rate; (Pa), partial pressure.

### Model Background

Following the enzyme-kinetic model and MME (Michaelis and Menten, 1913), we developed a novel enzyme-driven model (EDM) to determine how individual leaf photosynthesis responds to limited nutrients for both the C_3_ and C_4_ crop plants. It was tested using the data of the leaf photosynthetic rate responses to the [N]. Therefore, the relative changes of individual photosynthesis (𝜕*P*) should be constrained by the activity of the various basic enzymes (𝜕*vp*) of photosynthesis in crop plants at the leaf level (Nagaraj et al., 2017), at a time when enzyme activities were a limiting factor and the other conditions were constant:

 (1) $$\frac{\partial P}{p}\propto \partial vp,$$

where *P* represents the photosynthetic rate of the crops plants, *vp* denotes the activities of the enzymes associated with the photosynthetic rate of the crop plants, 𝜕*P* represents the differential of *P*, while 𝜕*vp* represents the differential of the enzymatic activity of photosynthesis. Hence, the relationships between the photosynthesis and enzyme activities were determined by combining this information with Eq. 1 as follows:

 (2) $$P\leq \;\propto e^{bvp},$$

where the coefficient of the transformation is represented by α, and *b* shows the potential of the enzyme activities (*vp*) in the crop plants. Therefore, Eq. 2 predicted that the photosynthetic rate increased exponentially with the enzymatic activity. Here, logarithmic (log) photosynthesis rates (ln *P*) and effective limited N sources (*R – R_0_*) were determined by applying log into Eq. 2, following the MME and letting the concentration of the nitrogen substrate ([S]) be proportional to the effective limited N resources (*R – R_0_*),

 (3) $$\ln P\leq \frac{\ln P_{max}(R-R_{0})}{K_{p}+(R-R_{0})}+\ln\;P_{0}$$

where ln *P_max_* = b*V_max_* and [*S*] = *R* – *R*_0_. Kp is the half Michael’s constant (ln P = P_*max*/2), *R* represents the limiting N concentration, and *R*_0_ presents the value of R, where ln *P* = ln *P*_0_, while *P_0_* is the coefficient of transformation (α) in Eq. 2, which is the photosynthetic rate when the effective resource (*R – R_0_*) is 0. This effective resource was presented in a lowest amount of stored [N] presented by the (P_0_ or P_store_) and used in the photosynthetic process of leaf production. Equation 3 predicts that the logarithm of the photosynthetic rate is dependent on the limited effective N content. Furthermore, by ignoring *P_0_* in Eq. 3, the response of the assimilation rates to the leaf [N] in the C_3_ and C_4_ plants (**Figure 3**) and the response of the photosynthesis rates to the current and high partial pressures of [CO_2_] with regard to the leaf N contents in the C_4_ and C_3_ plants, were also tested (**Figure 4**).

### Statistical Analysis

All statistical analyses were implemented in Origin 9.0 software (Data analysis and Graphing software). All the data (figures and tables) were tested using one-way ANOVA and Tukey’s test to assess each of the parameters. Data were log-transformed before analysis with Eq. 3. Most data were obtained from the supplementary resources of previous publications. These data sources were collected to verify our predictions by fitting exponential regressions between individual photosynthesis rates and the enzyme activities of the C_3_ and C_4_ plants in young leaves (**Figure 1**). Standard errors are given in parentheses (**Table 1**). A nonlinear correlation was used (Eq. 3) to evaluate the relationships between the various C_3_ and C_4_ plants. Furthermore, the assimilation rates for the same amount of leaf N were tested in C_4_ (maize) and C_3_ (rice) plants. Finally, the rates of photosynthesis per unit leaf N content in the young leaves of C_4_ and C_3_ plants, at low and elevated atmospheric partial pressures of CO_2_ (36 and 100 Pa of CO_2_) were investigated.

## Results

### The Photosynthetic Rate Increased Exponentially With the Limiting Enzymatic Rate in Young Leaves

We applied our model on published literature to understand the response of photosynthesis to enzymes activity. Our model also explained the response of light saturated photosynthetic assimilation to changes in leaf nitrogen in various C_3_ and C_4_ plants. We also tested the crops plant to the leaf N content under low and elevated CO_2_ concentration.

Our predicted exponential model (Eq. 2) was the best fitting model, shown by the lowest Akaike’s information criterion (Akaike, 1973; Posada and Buckley, 2004) and the best R^2^, when compared with the linear equation (see **Supplementary Table 1** in **Supplementary Material**). Furthermore, the linear equation did not show the best feedback to photosynthesis, in comparison with our predicted exponential equation (EDM, Eq. 2). All the data were obtained under constant [N] conditions to show that the activities of certain key enzymes exponentially increased with the photosynthetic rate (*P*) at the leaf scale in C_3_ (wheat and rice) and C_4_ plants (maize and sorghum) (**Figure 1**) (see all data source in **Dataset_S1**). Conversely, the respective values for *P* were higher and had decreased enzymatic activities in wheat when compared with rice (**Figures 1A–D**). The lower enzyme activities increased *P* and indicated that along with the limiting enzymes, other limiting factors also affected the enzyme activities and *P* level. In contrast, the increased activity of PGA-kinase (**Figure 1B**) significantly enhanced *P* in wheat compared with rice. In addition (**Figure 1D**), lower activities of Rubisco also enhanced *P* in wheat than in rice. These results clearly showed that the Calvin cycle enzymes changed their activities in young leaves of different crops plant, which is also dependent on the leaf trait (Makino and Ueno, 2018) and leaf age (Yamaoka et al., 2016). The response of enzyme activities increased exponentially with *P* in young maize leaves. It appears that higher plants have a complicated mechanism, and the activity levels of the Calvin cycle enzymes are limited by other factors. Of these, the activity of three enzymes from the maize leaf, PCK, and NADP-ME, initially influenced *P*, whereas Rubisco activity initially enhanced and then reduced *P* when compared with effects of other enzymes such as PCK and NADP-ME (**Figures 1E–G**). Our exponential model showed that all the three enzymes exhibited a significant correlation with *P* (P < 0.05) compared to linear model (see in **Supplementary Material**; **Supplementary Table 1**). Compared with Rubisco, the PEPC displayed an increased *P*, whereas the Rubisco contributed to the higher enzymatic activities in sorghum (**Figures 1H, I**). The results posit that PEPC had a higher affinity to Rubisco, which led to an increased *P* in the sorghum leaf. Therefore, the data used for sorghum were taken from low to high N sources (Makino and Ueno, 2017; see **Supplementary Material** for **Supplementary Figure 1**), which showed that PEPC and Rubisco decreased their activities by reducing the concentrations of N. The results revealed that reducing the N supplies resulted in a reduced *P*, as shown in sorghum (see in **Supplementary Material** for **Supplementary Figure 1**). Along with the limited Calvin cycle enzyme activities and N limitations, some biochemical and physiological traits are indirectly involved in the interspecific differences of the *P*.

### Photosynthetic Rate Dependence on Leaf [N] at the Leaf Scale

We assessed 11 species, including nine C_3_ plants and C_4_ grasses (two C_4_ plants: sorghum and maize) for their photosynthetic capacities, to explain the higher use of photosynthetic N at the leaf level (**Figure 2**). Under saturated light conditions, the amount of leaf [N] was lower in both the C_4_ plants (two C_4_ plants) compared to C_3_ plants (nine C_3_ plants). However, the C_4_ plants had the highest maximum CO_2_ photosynthesis rate (*P*_max_) but the lowest amount of stored [N] (*P_0_*), when compared with the C_3_ plants (**Figures 2A–K**). Across all 11 plant species, the photosynthetic capacity was the highest in the C_4_ plants (**Figures 2A, B**) and the lowest in the C_3_ plants (**Figures 2C–K**). However, even when there were low N uptake concentrations (higher *P_max_* needs less N absorption), C_4_ plants had greater photosynthesis rates (*P*_max_) when compared with C_3_ plants (higher *P_max_* needs more N absorption). For instance, rice, maize, and sorghum showed the highest affinity (half-saturation constant, Kp, for leaf [N]), in decreasing order. However, both maize and sorghum exhibited the lowest *P_0_* (half to the highest *P*_max_) across all 11 plants. *P_0_* is the minimum amount of stored [N], mainly used for leaf expansion and photosynthetic capacity. Rice had the highest amount of leaf [N] or uptake after *Prunus persica*, and thus achieved the highest *P_max_* rate of all nine C_3_ species tested. Therefore, with the same amount of leaf N content, maize (C_4_) showed a higher *A*_rate_ relative to rice (C_3_) (**Figure 3**). Interestingly, *Prunus persica* achieved the highest leaf [N] but still showed the lowest *P_max_* for all 11 plants. These results showed that the allocation of N to the photosynthetic machinery decreased, but still enhanced the *P_max_* rate when compared with all the C_3_ plants.

### The Shift of the EDM From Low to Elevated Pa [CO_2_]

The responses of the C_3_ and C_4_ plants to the leaf N contents under 36 and 100 Pa [CO_2_] and the effects of the CO_2_ on the maximum photosynthetic rate (*P*_max_) and the uptake of the leaf N content were examined (**Figures 4A, B**). The results demonstrated that under both Pa [CO_2_] conditions, the maize plants maintained the maximum *P_max_* rate compared with the C_3_ plants. Interestingly, maize showed higher *P_max_* and absorption of leaf N content under elevated Pa [CO_2_] (**Figure 4B**). In the maize plant, the response of the Kp (half-photosynthesis constant) to the leaf N content showed a higher affinity under elevated CO_2_ (**Figure 4B**). Although while transgenic anti-rbcS 77 (wild rice) had 65% wild-type Rubisco, it still had a lower *P_max_* when compared with the maize under elevated Pa. Although these results suggest the suppression of *P_max_* and leaf N content under the elevated Pa when compared with the low Pa due to the enrichment of the CO_2_ in the rice plants (**Figures 4A, B**), the results suggest that long-term CO_2_ decreases the initial stimulation of photosynthesis and then down-regulates it; this finding suggests a decrease in the Rubisco content in plants. These results revealed that the leaf N content and Rubisco were closely related, which further directly or indirectly affects photosynthesis. Many researchers have suggested a positive correlation between photosynthesis rates and leaf N content, and we have validated this by showing that N deficiency causes a reduction in the photosynthetic rate and intercellular CO_2_ concentrations (Ci) (see **Supplementary Figure 2** in **Supplementary Materials**). Thus, N deficiency and CO_2_ concentrations are important for crops in the future, both physiologically and morphologically.

## Discussion

### Photosynthesis Rate Showed Exponential Response to Enzyme Activities

We made a simple prediction, that the photosynthetic rate (*P*) of various plant species would be scaled non-linearly in relation to their enzyme dynamics and limited leaf [N]. Previous studies showed that activities of the Calvin cycle enzymes changed slightly from the young to the mature leaves of rice (Yamaoka et al., 2016), and in wheat (Suzuki et al., 1987). In addition, there were other limiting factors that inhibited enzyme activities and affected *P* in the crop plants. In our model, we found lower activities of cpFBPase still enhanced higher *P* in wheat than in higher activities of cpFBPase with lower *P* in rice (**Figure 1C**). This higher linear correlation of cpFBPase with the CO_2_ photosynthesis rate in wheat, had previously been reported (Sudo et al., 2003). A positive correlation was also found between the Calvin cycle enzymes such as cpFBPase and the photosynthesis rates (Miyagawa et al., 2001). While Rubisco showed lower activity, it still enhanced *P* in wheat than in rice. This showed that wheat exhibited a higher capacity for the assimilation of C when compared with rice. Earlier reports mentioned that changes in the amount of N supplied directly, enhanced the rate of leaf expansion progressively and therefore enhanced the capacity for C assimilation, which correlated with the increased levels of key photosynthetic enzymes (Huber et al., 1989). In certain conditions, rice was revealed to have higher photosynthetic rates, due to the higher conductance of CO_2_ through the cell wall, chloroplast thickness, and carbonic anhydrase activity, when compared with wheat (Makino et al., 1988). Moreover, the synthesis of Rubisco decreased with advancing leaf maturity, which was closely interlinked with decreases in the N influx into the leaf, as demonstrated by Imai et al. (2005). This suggests a higher RUBP regeneration capacity in wheat when compared with rice. The results (**Figures 1A–D**) revealed that the Calvin cycle enzymes in wheat and rice plants responded differently, clearly showing variations with leaf traits and developmental stages. Rubisco synthesis was completed during the leaf expansion, while the concentrations of the Rubisco regulated the levels of protein degradation during the leaf senescence (Mae et al., 1984: Makino et al., 1984: Suzuki et al., 2001). This indicates that the synthesis of Rubisco could be stimulated if a greater N concentration was present in the senescing leaf, because N influx drops during leaf senescence and is closely associated with the synthesis of Rubisco (Imai et al., 2008). These variations in the activities of the limited Calvin cycle enzymes depend on leaf age (Prasad et al., 2009), and leaf ontogeny (Sesták, 2012). Similar results were reported and explained for various key enzymes that were correlated with the photosynthetic activities of C_4_ species (Usuda et al., 1984) and the changes associated with leaf age in maize (Usuda, 1984), and various levels of N (Sugiyama et al., 1984). Therefore, we tested our exponential model where the enzyme activities showed higher significance (*P* value and R^2^) and lowest AIC values compared to the linear model (See **Supplementary Material** for **Supplementary Table 1**).

On the contrary, *P* showed positive exponential correlations with the activities of NADP-ME, PCK, and Rubisco (**Figures 1E–G**). Similarly, a positive correlation was also found for the photosynthesis rate and activity of NADP-ME (Nose et al., 1994). Furthermore, PCK activity increased, leading to higher photosynthesis rates and a higher exponential correlation with *P* (**Figure 1F**). The PCK enzyme is highly expressed in the BS cells, where oxaloacetate releases CO_2_, which is fixed through Rubisco. Although, NADP-ME activity showed the lowest activity, it still enhanced the *P* in maize. Previous results reports the reason that in the C_4_ cycle, NADP-ME was involved in short steps of the enzyme and amino acid pathways (Kanai and Edwards, 1999), and tended to have higher PNUE and NUE (Ghannoum et al., 2005). Therefore, various characteristic traits play important roles in the photosynthetic rate and could be regulated by the electron transport rates in maize. Some studies like NADP-ME in maize, and Rubisco were also involved in the re-fixation or regenerate phosphoenolpyruate carboxylase (PEPC), and as a result Rubsico is a rate-limiting enzyme (Yabiku and Ueno, 2017), which has positive correlations with the photosynthetic rates (Baer and Schrader, 1985). PEPC did not show a correlation with the photosynthesis in maize (Yabiku and Ueno, 2017), but showed a significant relationship in sorghum (Makino and Ueno, 2018). Various studies suggest that higher NUE occurred due to various genetic factors that were closely related to the N content in maize (Yabiku and Ueno, 2017). However, our model suggest that the exponential relationship between NADP-ME activity and *P* possibly be differ among species.

The highest positive correlation with *P* was in the leaves of sorghum; however, it had higher Rubisco enzyme activity (**Figures 1H–I**). PEPC exhibited higher affinity and was strongly correlated to *P* in C_4_ plants, when compared with the C_3_ plants, and followed the EDM. As the data were taken from low to high N sources, the ratio of the PEPC and Rubisco declined with the reducing N contents. Compared with the Rubisco, the PEPC showed greater reductions in line with the reduced N content at the leaf level (Makino and Ueno, 2018), and similar responses were also found in *Amaranthus retroflexus* (Sage et al., 1987) and maize (Sugiyama et al., 1984). Therefore, C_4_ plants with lower enzyme levels revealed a higher affinity of PEPC for CO_2_ to achieve higher *P*. A strong positive correlation is commonly observed between both PEPC and Rubisco activity and maximum photosynthetic rates (Von Caemmerer et al., 1997). Similarly, in maize, it was reported that N was partitioned into PEPC, which may function as a storage reservoir for excess leaf N (Uribelarrea et al., 2009), and consequently, the optimum maximum growth was exceeded (Sugiyama et al., 1984; Makino et al., 2003). In response to the supply of N (Suzuki et al., 1994), the PEPC activity increased with the N supply.

Therefore, N plays a huge role alongside enzymes in increasing the *P*. Similarly, reducing the N supply resulted in a reduced photosynthesis, N content, chlorophyll content, and PEPC and Rubisco carboxylase/oxygenase activity. Therefore, our model predicts that increasing the N content per leaf area would enhance the thylakoid and Calvin cycle enzymes, which would change the key physiological processes. This finding has a fundamental application for their N utilization and N uptake in that enzyme activities play a role in the rate of plant growth and photosynthesis. Most enzyme activities showed a higher coefficient of correlation (**Figure 1** and **Table 1**); therefore, we have shown that enzyme dynamics possibly drive the photosynthesis rate and modeling of resource fluxes. The main finding of our research is that our model explains the exponential relationships very significantly. Therefore, our model were tested and fitted compared to linear model. We tested our exponential model where the enzyme activities showed higher significance (*P* value and R^2^) and lowest AIC values compared to the linear model (**Table 1** and see **Supplementary Table 1** in **Supplementary Material**).

### Response of Photosynthetic Rate Dependent on Leaf Nitrogen Content

Some plant species require various N sources to regulate photosynthesis (Evans, 1989; Rotundo and Cipriotti, 2017). This study demonstrates that this variation is a determinant of the amount of leaf [N] under saturated light conditions and exhibits the response of Kp (half-saturation constant) to leaf [N] (**Figures 2A–K**). C_4_ species (maize and Sorghum) showed the highest affinity to leaf [N], which contributed to their having the maximum photosynthesis rate (*P_max_*) (**Figures 2A, B**). Accordingly, for each plants N allocation to the leaves, an optimum N content exists to maximize its crop biomass production (Sinclair and Horie, 1989). A higher PNUE was observed in maize plants with efficient use of N, to increase their NUE and biomass production (Mu et al., 2016). In this study, it was found that C_4_ species could enhance their physiological NUE if N storage rates were lowered by enhancing *P_max_*, which is the PNUE. Thus, C_4_ species that invest less N greatly enhanced their *P_max_*, which is in accord with previous studies that C_4_ species exhibit higher *P_max_* because of the speed of the Rubisco, which enables them to invest fewer N resources into Rubisco (Young et al., 2016). With the same amount of leaf N content (Evans and von Caemmerer, 2000), maize clearly showed assimilation rates higher than rice. Therefore, maize showed higher N assimilation and higher NUE than the rice (**Figure 3**). C_4_ species have a higher ability to utilize CCM to concentrate CO_2_ around Rubisco and suppress photorespiration and RUBP regeneration (Sage and Zhu, 2011). Previous studies showed that in C_4_ plants, higher amounts of N investment into the thylakoids could possibly maintain greater NUE (Makino et al., 2003). Thus the present results revealed that every plant species showed substantial differences in leaf N content as a result variance in *P_max_*. That’s why the differences in plant leaf *P_max_* among the C_4_ and C_3_ species possibly attribute to differences in physiological and biochemical features in their leaves.

C_3_ plants (**Figures 2C–J**) have larger *P_0_* than did C_4_ plants (**Figures 2A, B**); for this reason, C_3_ species still require more N to get a higher *P_max_.* Here, we can evaluate a leaf physiology that both C_4_ plants invest less N content in leaf production to enhance higher *P_max_* compared to invest more N required to enhance photosynthesis in C_3_ plants. Similarly, the previous results have shown a higher N content used in the photosynthetic processes of C_3_ plants (Evans and Seemann, 1989; Evans, 1989). Unexpectedly, the stored N in the form of *P_0_* was also higher in the C_3_ species than in the C_4_ species. C_4_ species, even at low *P_0_*, can maintain a higher *P_max_* and a higher probability of survival than can C_3_ species. Hence, nutrient stoichiometry changes due to the availability of N; such variations enable plants to increase their C uptake and enhance the efficiency of using their resources to fix C under both C- and N-limiting conditions for plant growth. As C_4_ species have a higher importance because of their enzyme activities, and their various resource allocations, as a results, C_4_ photosynthetic pathways can invest more N into leaf production, than C_3_ pathways. Our model from the analysis revealed that allocation of N in C_4_ species is higher into leaf thylakoid and invest less N into Rubisco than in C_3_ plants.

In our model, photosynthetic rate of C_4_ species showed greater dependence on N content and light, than the nine C_3_ species assessed; consequently, these results determined greater NUE in the C_4_ species than the C_3_ species. Evolutionary pressures appear to have concentrated the enzymes towards more efficient utilization of CO_2_ (Jordan and Ogren, 1981). Consequently, the evolution of plants from C_3_ to C_4_ is marked by the limitation of photorespiration (Kelly, 2018), which requires a high level of CO_2_ concentrated around the Rubisco. Previous studies demonstrated that *Amaranthus retroflexus* showed greater NUE than *Chenopodium album* (Sage and Pearcy, 1987). In addition, the diversity of the photosynthetic capacity, which is correlated with leaf traits, explained the relative allocations of N to the photosynthetic functions and showed various PNUE among the different crop plants.

Across all C_3_ plants (**Figures 2C–K**), rice (**Figure 2D**) achieved higher leaf N content and a higher *P_max_*. This study demonstrated that across various C_3_ species (**Figures 2C–K**), rice showed the lowest stored *P_0_* after C_4_ species such as maize and sorghum (**Figures 2A, B**). Therefore, to get maximum *P_max_* rates, rice needs a lower amount of stored N in the form of *P_0_*. The results of this study revealed that the *P_max_* rate may be enhanced when more N is supplied. Storage N and various residues of N could be enhanced in the leaves due to the supplies of N (Yasumura and Ishida, 2011; Avice and Etienne, 2014; Wyka et al., 2016). Photosynthesis proteins and young tissues use the stored N for their growth. Under low N conditions, young leaves need a higher supply of N, which leads to decreases in the stored N pool size in mature leaves (Liu et al., 2018). In rice, which showed the highest *P_max_*, increased carbonic anhydrase activity appears to have a direct relationship with the mesophyll conductance (Makino et al., 1992), which is closely related to the surface area of the chloroplast (Terashima et al., 2005). Our model posits that the amount of N affects leaf thickness, which strongly affects mesophyll conductance and causes variations in the nutrient cycle and plant growth capacity. Thus, higher amounts of N and Rubisco both functioned as stored N proteins and catalytic enzymes, respectively. This was similar to the findings of Warren et al. (2003), according to which, with increasing N concentrations, Rubisco functions as a storage protein in *Pinus sylvestris*. Such limitations of the leaf N content affect the proteins involved in the Calvin cycle, resulting in the described photosynthetic regulation.

The PNUE is an important leaf trait that describes adaptive strategies, physiology, and the leaf economics of a species (Onoda et al., 2017) and may indirectly reflect the efficiency of the N utilization (Feng et al., 2008). Most importantly, our results demonstrated that even at low N, C_4_ species still had higher PNUE, causing higher N allocations, and the upregulation of photosynthesis and increases in C gain. Therefore, C_4_ species revealed higher C gains than did C_3_ species. Both the C and N levels control leaf expansion (Lattanzi et al., 2005; Pantin et al., 2011). The higher N content present because of the limited C in the leaves, enhances the capacity of leaf photosynthesis (Liu et al., 2018). Similarly, Rubisco and PEPC activities vary with N nutrition, leaf age, and light intensity during plant growth (Usuda, 1984; Meinzer and Saliendra, 1997). This explains why the photosynthesis rate depends on the N sources, storage N, and species-specific photosynthetic capacity characteristics. At last, the variance response of leaf physiology can be evaluate or judge by the variance in PNUE, leaf N content and N allocation in C_3_ and C_4_ plants, explained by our model; which is also explained by other studies (Poorter and Evans, 1998; Westbeek et al., 1999). Therefore, the results reveal the generality of the impact of nitrogen allocation to the interspecific difference in PNUE.

### Response of Photosynthetic Rate to Nitrogen Content Under Low and Elevated Pa [CO_2_]

Our model clearly shows that maize exhibited the highest *P_max_* under elevated 100 Pa [CO_2_] (**Figure 4B**). As shown in **Figures 4A, B**, however, the leaf N content was affected by the eCO_2_; however the *P_max_* rate decreased with eCO_2_ in rice. On the other hand, anti-rbcS 77 (*rbcS* antisense rice with 65% wild-type Rubisco) under eCO_2_ enhanced N content as a results increased *P_max_* than in rice (**Figure 4B**). The results revealed that in transgenic rice of anti-rbcS 77, the reduced activity of Rubisco leads to reallocation of N in to leaf thylakoid as a results increased NUE, which is in accord with the earlier study (Makino et al., 1997). Then the increased NUE suggests that anti-rbcS 77 invest more N into photosynthetic apparatus that are involved in *P_max_*. While on other hand, the result clearly predicts a decrease in the *P_max_* of rice at elevated Pa, because the CO_2_ enrichment was correlated with starch accumulation in the plant leaf blades (Nakano et al., 1997), which caused reduction in leaf N content. Thus, the decrease in the photosynthetic capacity could possibly be related to a reduction in the leaf N content. Rice (C_3_ plant) showed the lowest leaf N content by low [CO_2_] compared with *Spinacia oleraceae* and *Phaseolus vulgaris* (C_3_ plants); however, the enhanced maximum *P_max_* was comparable across all C_3_ plants. Rice maintained higher N utilization in the leaves, when compared with the other C_3_ plants, under low eCO_2_ (**Figure 4A**). This indicates that the reallocation of N can provide a mechanism to enhance the biomass and *P_max_* rate, as described by Field (1983), thus enhancing the whole-plant C gain. Higher N allocation enhances the PNUE in the photosynthesis process with higher supplies of N (Hikosaka and Hirose, 1998). In general, high photosynthetic rates lead to enhanced growth rates and maintenance rates. Therefore, Farquhar et al. (1980) developed a photosynthetic model for the C_3_ pathway, a photosynthesis rate limited by Rubisco at low PaCO_2_ and by electron transport capacity at high PaCO_2_. The varying Kp responses of the leaf N to CO_2_ among the different plants, indicated that the photosynthetic responses of the plants to the low and elevated CO_2_ were not only due to the levels of the CO_2_ but also the N contents. However, every species has a different photosynthetic capacity to store a high N source and then to utilize it for their maximum performance for new tissue and plant growth. Thus, the reduction in the N content in the leaf is a known indicator of photosynthetic accommodation to eCO_2_ (Leakey et al., 2009).

N deficiency reduces the size of the chloroplasts (Bondada and Syvertsen, 2005), and thus high levels of stored N may increase the size of the chloroplasts in rice cultivars (Laza et al., 1993); with higher chloroplast sizes being beneficial for higher C gains. In addition, our model showed that the N was influence by high PaCO_2_ in maize and rice (**Figure 4B** and **Table 2**), which directly or indirectly affects the size of the chloroplasts (Bondada and Syvertsen, 2005), because the chloroplast morphology and ultrastructures affect the photosynthesis. Similar results reported that long-term elevated CO_2_ usually causes a reduction in the photosynthetic capacity, as it is directly related to the decreased levels of Rubisco and other C_3_ Calvin cycle enzymes (Ghannoum et al., 2000). Although sorghum (C_4_) uses N more efficiently than most C_3_ plants, N deficiency suppressed the Ci and photosynthesis (see **Supplementary Figure 2** in **Supplementary Material**). Maranville and Madhavan (2002) reported that N deficiencies also decreased the levels of both Rubisco and PEPC in sorghum leaves. Like some species that have the C_3_ photosynthetic pathways, Rubisco is likely to represent the single remobilized reserve of protein-N, which generally accounts for 30%–60% of the total soluble protein, 20%–30% of the total leaf N in C_3_ plants (Makino, 2003), and 5%–9% of the total leaf N in C_4_ plants (Sage et al., 1987). However, maize still had a high *P_max_* due to the variance in the regulation of the photosynthetic C_4_ gene expression (Sheen, 1999).

Our findings also support and show that Rubisco activity is closely related to the low affinity of CO_2_ (Sage, 2002); thus, a lower level of Rubisco is enough to enhance the *P_max_* in maize plants. Interestingly, the published data predicted that even maize exhibited a low affinity for *P_max_* rates, but higher N levels showed that more allocations of N had occurred, and sometimes, Rubisco acted as a storage protein over a long period to enhance the growth and biomass production for a long leaf lifespan, under elevated PaCO_2_. Besides, N content is enhanced in the maize leaves with eCO_2_ and suggests that elevated CO_2_ increased leaf N metabolism and amino acid biosynthesis. Similar results are also reported in root N metabolism (Cohen et al., 2019a). In rice species, leaf [N] reduced from low to elevated CO_2_ (**Figures 4A, B**), suggesting that higher investments of the N in Rubisco caused less N to be invested in the PNUE and storage proteins. Consequently, a high amount of leaf N is directed to the biosynthesis of the photosynthetic machinery. From these observations, elevated CO_2_ influences the limitations in the photosynthetic capacity to decrease N allocations to proteins and Rubisco that are involved in electron transport (Liberloo et al., 2005); hence, the reduction in N allocation possibly serves to enhance available and mobile N for new foliage growth. Hence, results support the reallocation of N among crop plants, which could provide an adaptation mechanism in response to climatic changes, and rising atmCO_2_ concentrations may improve the physiological process model.

Rubisco expression was adversely affected by elevated CO_2_ in tomato plants (Cohen et al., 2019a), which suggests that the reduced uptake rate of N occurred due to the atmCO_2_ concentrations in the leaves of C_3_ plants, and thus, due to the substantial rise in PNUE. The low leaf N concentration under the eCO_2_ in rice (C_3_ plant; **Figure 4B**), suggests a lower allocation of N to photosynthetic apparatus which is important determinant of PNUE. Thus, our model showed that eCO_2_ affect the N concentration in rice as a results reduced the *P_max_*. Thus, a higher NUE could increase the allocation of N to the photosynthetic process due to the higher N availability (Poorter and Evans, 1998). The response of crops to eCO_2_ (Long et al., 2006) revealed that crop plants under elevated CO_2_ decreased the activity of Rubisco for the regeneration of RUBP. Since in a C_4_ plant, a huge utilization of the N occurs in the thylakoid, the CO_2_ concentrating process maintains greater NUE for photosynthesis (Makino et al., 2003). In this case, the results support that under the various levels of PaCO_2_, the positive relationships found between the physiology and N content, may be useful in various responses of the photosynthesis in crop plants. Therefore, the reduction in the amount of Rubisco would be an advantage to enhance NUE. Therefore, the high CO_2_ emissions worldwide and global warming, have intense impacts on important crop plants, their production, and their dynamic metabolic balances, particularly at the leaf level.

## Conclusions

The different exponential responses of the key enzyme activities to the photosynthesis rates clearly showed leaf trait variations and the developmental stages of the leaves for wheat and rice. In the young leaves of wheat and rice, the enzyme activities exponentially increased with the photosynthetic rate. Our findings suggest that enzyme activities and photosynthesis rate showed higher exponential correlation than linear correlation. In the young leaves of maize, PCK, NADP-ME, and Rubisco were positively correlated with the photosynthetic rates. In sorghum, it was suggested that both the PEPC and Rubisco increased with the increased leaf N content. All enzymes exhibited higher exponential relations with photosynthesis compared to linear relations. C_4_ plants (sorghum and maize) exhibited higher affinities to light and N sources than C_3_ plants; therefore, we identified that C_4_ plant species had a higher PNUE and higher C gain. Regarding leaf economy, the stored N source provided half of the N required for the maximum photosynthetic capacity used for leaf expansion and plant growth. Our findings suggest that the Kp affinity of various species is a key indicator for various species, which affects resource allocation. Finally, the elevated CO_2_ had a negative effect on the N concentrations of C_3_ plant leaves and needs to be investigated further in more crop plants.

## Data Availability Statement

All datasets generated for this study are included in the article/**Supplementary Material**.

## Author Contributions

AK and GW designed the research. AK and LL collected the data. AK, LH, and LL analyzed the data. KX and HH designed **Figures 1**, **2** in Adobe Illustrator CC. AK, KX, and GW wrote the manuscript.

## Funding

This work was supported by the National Natural Science Fund of China (31330010) and Natural Science Foundation of Zhejiang Province (LZ13C030002).

## Conflict of Interest

The authors declare that the research was conducted in the absence of any commercial or financial relationships that could be construed as a potential conflict of interest.
