# Supplementary material for: Validation of an Enzyme-Driven Model Explaining Photosynthetic Rate Responses to Limited Nitrogen in Crop Plants
Source: Front Plant Sci. 2020 Sep 25;11:533341. doi: 10.3389/fpls.2020.533341 (PMC7546270; doi:10.3389/fpls.2020.533341)
Supplement: Supplementary file 1 [file DataSheet_1.docx]

**Supplementary Material**

**Supplementary Tables and Figures**

**Supplementary Table 1**

| **Supplementary Table 1.** Comparison of an exponential equation and linear equation model for key-enzyme activities and photosynthesis rates.  Model | | | | | | | | |
| --- | --- | --- | --- | --- | --- | --- | --- | --- |
| Key-enzymes | **Exp. Eq.** | | **Linear Eq.** | | **Exp. Eq.** | | **Linear Eq.** | |
|  | *AIC* | R^2^ | *AIC* | R^2^ | *AIC* | R^2^ | *AIC* | R^2^ |
|  | ***Triticum aestivum*** | | | | ***Oryza sativa*** | | | |
| NADP-G3PDH | 134.94 | 0.857 | 146.72 | 0.816 | 27.66 | 0.921 | 42.82 | 0.932 |
| PGA Kinase | 30.70 | 0.981 | 49.83 | 0.981 | 20.77 | 0.968 | 51.46 | 0.929 |
| cpFBPase | 39.04 | 0.984 | 80.31 | 0.966 | 23.36 | 0.929 | 41.40 | 0.922 |
|  | ***Zea mays*** |  |  |  |  |  |  |  |
| NADP-ME | 29.05 | 0.828 | 142.67 | 0.24 |  |  |  |  |
| PCK | 42.25 | 0.839 | 144.57 | 0.321 |  |  |  |  |
| Rubisco | 33.83 | 0.851 | 146.31 | 0.346 |  |  |  |  |
|  | ***Sorghum bicolor*** |  |  |  |  |  |  |  |
| Rubisco | 42.63 | 0.87 | 78.97 | 0.597 |  |  |  |  |
| PEPC | 27.84 | 0.953 | 74.02 | 0.79 |  |  |  |  |
| **Note:** Akaike’s information criterion (*AIC*) and best R^2^ were used. | | | | | | | | |

**Supplementary Figure 1**

**
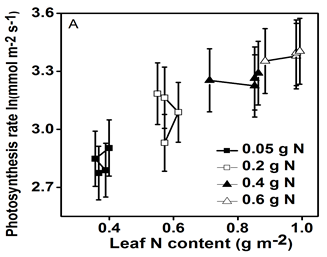
**

**Supplementary Figure 1.** Relationships in *Sorghum bicolor* between the photosynthesis rates and leaf N contents (0.05, 0.2, 0.4, or 0.6 g N). Photosynthesis rate increased with increased amount of leaf N content in sorghum, respectively. The error bars indicate the standard errors. Data taken from (Makino and Ueno 2018).

**Supplementary Figure 2**

**
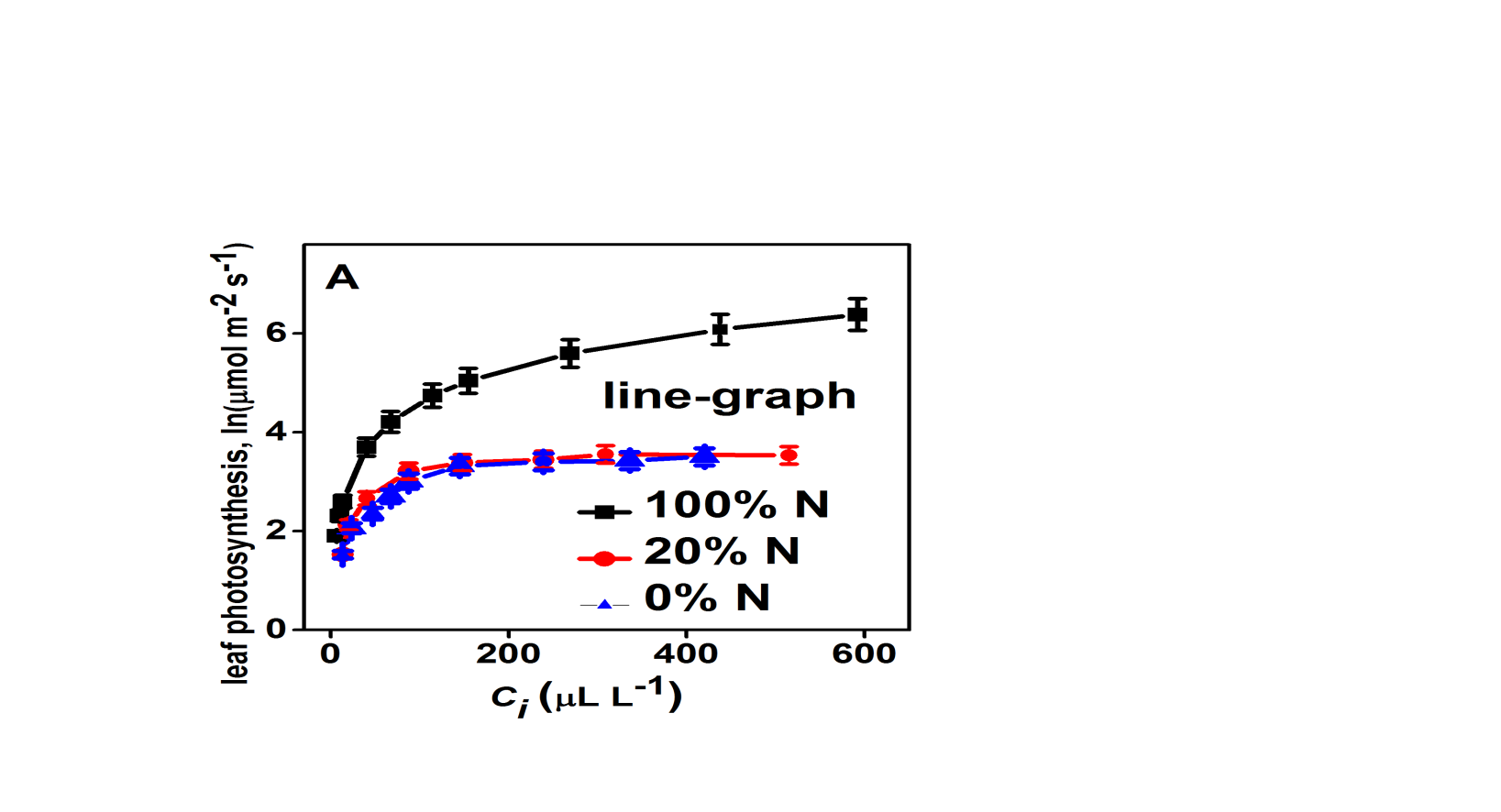
**

**Supplementary Figure 2.** Response of the leaf photosynthesis rates on the intercellular CO_2_ concentrations (*C_i_*), due to the nitrogen deficiencies in the *Sorghum bicolor*. Three types of Hoagland nutrient solution (0, 20, and 100 %) were tested, and after 46 to 50 days measurements were taken of the fully expanded leaves. The nitrogen deficiency in the leaves is seen to cause a decrease in the *C_i_*. While, the 0 and 20 % N reduced the leaf photosynthesis rates, they also reduced the intercellular CO_2_ concentrations, compared with the 100 % N. The error bars indicate the standard errors of three measurements. Data are taken from (Zhao et al, 2005).
